# Understanding AI adoption through expert discourse: A UTAUT-based analysis on LinkedIn

**DOI:** 10.1371/journal.pone.0344013

**Published:** 2026-04-16

**Authors:** Ali Yari, Mohammad Taghi Taghavifard, Iman Raeesi Vanani

**Affiliations:** Department of Operations Management and Information Technology, Allameh Tabataba’i University, Tehran, Iran; MCC Boyd Tandon School of Business, INDIA

## Abstract

Technology experts shape, rather than follow, the trajectory of artificial intelligence (AI). Yet their collective voice on professional social networks has been largely unmapped. Drawing on tens of thousands of AI-related LinkedIn posts, this study marries an embedding-based topic-modeling pipeline with the Unified Theory of Acceptance and Use of Technology (UTAUT) to decode what those who build AI really value. Our findings move beyond a simple application of UTAUT to re-contextualize its core constructs for this expert population. We find that for these experts, adoption is a complex negotiation: Performance Expectancy (PE) is redefined as the potential for industry-wide transformative breakthroughs, Effort Expectancy (EE) evolves into a demand for cognitive efficiency, Social Influence (SI) becomes a dual role where experts both shape and are shaped by norms, and Facilitating Conditions (FCs) are viewed as a holistic ecosystem. Furthermore, our analysis shows how cultural context recalibrates each construct, underscoring that “one-size-fits-all” models misread global AI uptake. Beyond mapping discourse, this study delivers actionable foresight for leaders navigating the next wave of AI innovation. Based on the analysis of expert discourse, we proposed a re-contextualized UTAUT model for AI adoption, as illustrated in the conceptual model.

## 1. Introduction

Artificial Intelligence has rapidly evolved from a specialized academic field into a transformative force reshaping global industries. This evolution, fueled by advancements in machine learning, big data, and cloud computing [1,2]This has spurred unprecedented innovation, particularly within the high-tech sector. In this dynamic landscape, understanding the mechanisms of technology adoption is paramount. While much research has focused on general user perceptions, the discourse of technology experts—the developers, researchers, and thought leaders who build and champion these technologies—offers a uniquely valuable and forward-looking perspective. These experts are critical agents whose knowledge significantly enhances strategic decision-making and organizational performance. [3,4].

Despite their pivotal role, the collective perspective of these experts as expressed on professional social networks remains a relatively underexplored area in the technology acceptance literature. While platforms like LinkedIn serve as a primary venue for professional knowledge exchange and discourse [5] the sheer volume and unstructured nature of this data present a significant analytical challenge. This study aims to fill this gap by systematically analyzing the large-scale discourse of technology experts to understand the key topics and underlying drivers of their engagement with AI.

To achieve this, we employ a mixed-method approach. First, we utilize topic modeling, a Natural Language Processing (NLP) technique, to identify the salient topics of discussion from a large dataset of posts. NLP tools are increasingly used to process and extract relevant information from large volumes of social media data [6,7]. Second, we use the UTAUT as a robust theoretical lens to analyze and interpret these topics [8]. This approach allows us to move beyond anecdotal evidence and build an empirically grounded model of AI acceptance from the perspective of its core professional community.

This study proceeds as follows. First, we review the UTAUT framework and identify a critical gap concerning its application to unsolicited expert discourse. Next, we detail our computational methodology for analyzing large-scale LinkedIn data. We then present the results of our topic modeling. Finally, based on these findings, we present a re-contextualized model of UTAUT, demonstrating how expert discourse expands and challenges the theory’s core constructs, and discuss the theoretical and practical implications.

## 2. Literature review

### 2.1. AI Adoption

Artificial intelligence adoption is shaped by factors operating at individual, organizational, and societal levels. At the personal level, key determinants include trust, security concerns, perceived ease of use, utilitarian benefits, and SI [9]. Among these, the most critical factors are perceived benefits of AI, system capabilities, and perceived compatibility [10]. Organizational factors encompass top management support, technical competencies, strategic road mapping, digital maturity, IT infrastructure, and the quality of the data ecosystem [9]. Additionally, cost-effectiveness, resource availability, and AI strategic alignment all play significant roles in shaping adoption intentions. Environmental influences, such as competitive pressure, vendor support, and government regulation, also affect adoption, while complexity and regulatory constraints can act as barriers [11]. Systematic reviews categorize these influences across technological, social, organizational, and environmental dimensions, highlighting the interconnected nature of factors at both firm and individual levels [12].

Across various contexts, AI adoption faces consistent barriers and enablers that span multiple sectors. Major obstacles include financial constraints, inadequate IT infrastructure, organizational resistance to change, and ethical concerns related to data privacy and algorithmic bias [13]. In healthcare settings, allied health professionals highlight a lack of AI knowledge, explainability challenges, and concerns about role replacement as significant barriers [14]. Additional challenges include bureaucratic hurdles, job security concerns, and accountability issues [15]. On the other hand, critical enablers involve visionary leadership, updated IT systems, and potential cost savings. Strong digital leadership, a supportive organizational culture, and advancements in NLP facilitate adoption in human resource management [16]. Overall, organizational preparedness, innovation-friendly cultures, and alignment with strategic business objectives emerge as fundamental success factors.

AI adoption differs from traditional technology adoption in several important ways. Trust requirements are more complex for AI, necessitating transparency, explainability, and certification, which are less critical for conventional technologies [17]. The adoption process itself is more challenging than previous information technology revolutions, with AI representing a particularly intricate implementation task [18]. Human capital plays a pivotal role, as pre-existing differences account for one-third to half of the variation in AI adoption across regions and industries [19]. Furthermore, AI diffusion follows distinct epidemic patterns characterized by clustering in industrial and regional hotspots, direct exposure to advanced AI knowledge sources, and relational embeddedness within AI knowledge networks [20]. This generates a relatively closed system of adopters that may restrict broader diffusion, in contrast to the more open patterns typical of traditional technologies.

Cultural, economic, and social differences also strongly influence AI adoption across regions and societies. Cultural identity shapes how individuals perceive AI systems: individualists often view AI as external threats to autonomy and privacy, whereas collectivists perceive AI as extensions of themselves that facilitate social conformity [21]. Regional variations are evident: Western countries emphasize privacy and ethical concerns, while African and Asian regions focus on technological dependency, state control, and socio-economic issues such as job displacement [22]. Cultural beliefs, attitudes, and organizational structures influence AI acceptance in organizations, resulting in adoption patterns that differ across Western, Eastern, and Global South paradigms [23]. In banking services, consumer adoption of AI technologies such as robo-advisory services varies across cultures, with differences linked more to social capital than to fear of novelty [24]. These observations highlight the importance of culturally-aware AI deployment strategies.

Organizations adopting AI experience shifts toward innovation, agility, and continuous learning. Nevertheless, implementation poses significant challenges. Employee resistance emerges as a consistent concern, alongside job security fears and inadequate training. Successful adoption requires effective leadership, transparent communication, and substantial investment in skill development [25].

### 2.2. The UTAUT theoretical framework

The Unified Theory of Acceptance and Use of Technology (UTAUT), developed by [8] paper, presents a comprehensive framework for explaining user acceptance of new technologies. The theory was formulated through a rigorous synthesis and consolidation of eight prominent prior models and theories in the field, including the Theory of Reasoned Action (TRA), the Technology Acceptance Model (TAM), the Theory of Planned Behavior (TPB), and the Diffusion of Innovation (DOI) theory. UTAUT identifies four core constructs as direct determinants of users’ behavioral intentions and subsequent use behavior.

The four core constructs of the theory are defined as follows:

#### 2.2.1. Performance expectancy.

This is the degree to which an individual believes that using the system will help them to attain gains in job performance. It stands as the strongest predictor of intention in the model and consolidates concepts such as perceived usefulness from TAM [26], extrinsic motivation, and relative advantage from DOI [27].

#### 2.2.2. Effort expectancy.

This is the degree of ease of use of the system. This construct captures the cognitive cost of interacting with a technology and is derived from concepts like perceived ease of use from TAM [26] and complexity. Its influence is most significant in the early stages of adoption.

#### 2.2.3. Social influence.

This is the degree to which an individual perceives those important others (e.g., colleagues, superiors) believe they should use the new system. It integrates concepts such as subjective norm from TRA [28] and TPB [29], and image from DOI [27].

#### 2.2.4. Facilitating condition.

This is the degree to which an individual believes that an organizational and technical infrastructure exists to support use of the system. It incorporates concepts like perceived behavioral control from TPB [29] and compatibility from DOI [27], and is unique in that it primarily predicts actual use behavior directly.

In addition to these core constructs, the original UTAUT model incorporates four key moderating variables **Age**, **Gender**, **Experience**, and **Voluntariness of Use**—that influence the strength of the relationships between the constructs and user intentions. In its foundational study, this comprehensive framework successfully explained approximately 70% of the variance in behavioral intention to use a technology.

It is important to note that the scope of this research was deliberately focused on the content of the expert discourse itself, rather than the demographic characteristics of individual participants. As such, the traditional UTAUT moderators age, gender, experience, and voluntariness of use were not incorporated into our analysis. This decision aligns with the study’s primary objective of mapping the collective topics of AI acceptance, leaving the investigation of individual-level differences to future research that could employ methods like surveys or interviews.

### 2.3. Application of UTAUT constructs in AI research

While the UTAUT framework provides a robust foundation, its core constructs have been extensively tested and adapted in the specific context of AI. The following review summarizes recent findings on how each of the four key constructs applies to AI adoption.

#### 2.3.1. Performance expectancy.

PE consistently emerges as the primary driver of behavioral intention to adopt AI. In studies based on the UTAUT model, this construct has successfully predicted technology acceptance among diverse groups, including librarians [30], passenger car sales personnel [31], and human resources professionals using AI-based HR information systems [32]. Evidence from meta-analyses confirms the significant positive effect of PE across various industries [33], which aligns with the original formulation of UTAUT [8] and the findings of the TAM regarding perceived usefulness [26]. This relationship is also clearly observed in the health sector [34]. However, some research reports that PE does not have a direct path to continuance intention [35]. Furthermore, user satisfaction is influenced by the confirmation of expectations and perceived usefulness [36]. AI users tend to adopt technologies that offer distinct functionalities compared to humans, and a violation of these expectations leads to a decrease in usage intention [37]. In this context, trust in AI also emerges as a critical factor that helps strengthen the relationship between PE and usage intention [32].

#### 2.3.2. Effort expectancy.

EE, referring to the perceived ease of use, is a key factor influencing the use of AI in various settings. This construct is significantly correlated with the use of AI-based recommender systems (Engström et al.). In education, teachers’ adoption of AI depends on multiple factors, including ease of use, self-efficacy, and perceived usefulness [38]. EE also has a considerable impact on the intention of healthcare providers to adopt AI [34], which is consistent with the initial trust of medical students in AI-based diagnoses [39]. In human resources, professionals’ willingness to use AI in recruitment depends not only on EE but also on perceived value and FCs [40]. Although the effect of EE on the intention to use AI coaching systems has been reported as weaker than PE and SI, it remains significant [41]. Moreover, demographic variables play an important role; for instance, younger individuals with higher economic status tend to have a more positive attitude towards this technology [42]. The effect of gender has also been observed in the application of AI in fintech [43] and AI-based health management [44]. Factors such as familiarity with technology and personal innovativeness also enhance AI adoption [43], whereas perceived privacy risks can have a negative effect [44].

#### 2.3.3. Social influence.

SI affects AI adoption through various pathways. Research shows that individuals’ education and income levels indirectly influence usage intention by increasing self-efficacy in using AI [37]. In the health sector, SI plays a mediating role between PE, EE, and the clinical intention to adopt AI diagnostic technologies [45]. Organizational culture is another key factor that increases employees’ readiness to use AI while reducing job insecurity stemming from it [46]. In human-AI teams, the AI agent itself can act as a social player, influencing human behavior by altering perceptions of control and justification [47]. Studies based on TAM have also repeatedly identified SI as a significant predictor of usage intention [33]. Similar research findings have been reported in the domains of healthcare [48], corporate social responsibility initiatives [49], and employee behavior at the firm level [12]. From a cultural perspective, a shift towards innovation facilitates AI adoption, while resistance to change and ethical concerns act as serious barriers [25].

#### 2.3.4. Facilitating conditions.

FCs, which include the necessary technical and organizational resources, are of paramount importance, though their impact depends on the specific context. In some cases, peer influence can play a more prominent role than individual attitudes [50]. For example, FCs directly guide farmers’ intentions to adopt AI in sustainable agriculture [51]. In engineering education, support from peers and leaders has increased faculty use and facilitated student exposure to technology [52]. Operations management studies also list FCs among the six key factors for technology implementation [53]. Analyses of necessary conditions show that FCs are vital for technology adoption; that is, without appropriate resources, adoption is not feasible, though their mere presence does not guarantee it, and their importance changes at different stages of adoption [54]. In radiology, practical factors such as financial pressures, the perceived value of AI, and the presence of a local champion play a significant role in facilitating AI adoption [55]. Organizational resources, such as senior management support and strategic alignment, also enhance technology adoption [11]. In public institutions, factors such as people, culture, structure, processes, and technology act as important facilitators [56]. While technology compatibility and management support directly promote adoption, competitive pressure has an indirect effect [57]. However, AI adoption may increase job stress, an effect that can be mitigated by coaching leadership, which underscores the need for supportive infrastructures [58].

In summary, while the existing literature confirms the relevance of UTAUT’s constructs, these studies are largely confined to specific user groups adopting technology within an organizational setting. A discernible gap remains in understanding how these constructs manifest in a dynamic, public ecosystem like a professional social network, where the ‘users’ are also the technology’s creators and shapers. This study addresses this gap by not merely applying UTAUT as a lens but by using expert discourse to explore the contemporary meaning and relevance of its constructs in the context of AI.

### 2.4. The research gap

While the literature on technology acceptance is extensive, a significant portion of it focuses on general users or specific organizational contexts. There remains a discernible gap in the large-scale, systematic analysis of the discourse produced by technology experts themselves. These individuals actively shape the narrative and direction of innovation, yet their collective perspective, as expressed in real-time on professional platforms, is a relatively underexplored area.

Beyond this phenomenological gap, there lies a significant theoretical one. Despite the widespread application and success of the UTAUT, the existing literature has predominantly relied on traditional self-report methods such as surveys and interviews. Consequently, our understanding of how the core UTAUT constructs—such as PE and SI—are manifested and articulated in the unsolicited, naturalistic discourse of these very experts remains significantly limited. This study, therefore, aims to fill this dual gap. Our objective is not only to map the focus areas of expert discourse but, more importantly, to examine how this discourse can inform, challenge, or re-contextualize our understanding of the UTAUT constructs themselves in the age of AI.

### 2.5. Research approach and questions

Professional social networks, particularly LinkedIn, have emerged as invaluable platforms for professional networking, knowledge exchange, and discourse among industry experts. The vast amount of unstructured text data generated on this platform provides a rich, dynamic repository of expert opinion. To analyze this data at scale, this study employs NLP, a field of AI that automates the processing and analysis of human language, enabling the identification of trends and the extraction of relevant information from large datasets.

By applying computational text analysis methods to the discourse of technology experts on LinkedIn, this research aims to move beyond anecdotal evidence and provide a structured understanding of their attitudes toward emerging AI technologies. The primary objective is to identify the key themes, points of focus, and underlying drivers of acceptance within this influential community. This approach allows for an empirical investigation into why some technologies gain traction and become central to the conversation, while others remain on the periphery.

Therefore, this study is guided by the following research questions:

- **RQ1: What are the primary topics discussed by technology experts regarding Artificial Intelligence on professional social networks?**
- **RQ2: How does an analysis of expert discourse inform, challenge, or re-contextualize the core constructs of the Unified Theory of Acceptance and Use of Technology (UTAUT) in the context of AI?**

By answering these questions, this research seeks to provide actionable insights for businesses, investors, and policymakers, illuminating the mechanisms of technology acceptance as defined by the very community that builds and champions it.

## 3. Methodology

**Fig 1:**
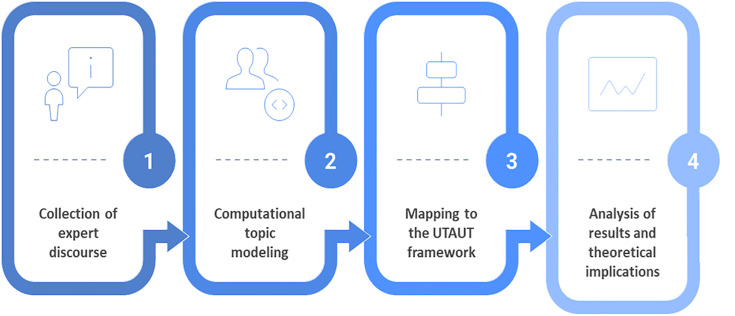
Research process stages.

This study follows a structured research design. The research process is presented in Fig 1.

### 3.1. Data collection and dataset description

**Fig 2:**
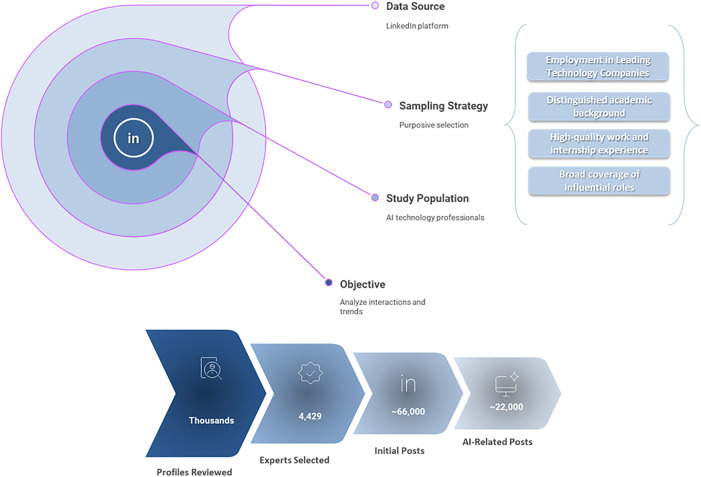
Data collection and dataset description.

The data collection procedure and dataset description are presented in Fig 2.

This study is based on a corpus of publicly available LinkedIn posts authored by professionals active in the AI domain. LinkedIn was selected as the empirical context because it functions as a professional discourse arena in which experts routinely share technical insights, strategic perspectives, and reflections on technology adoption across organizational and industrial settings.

#### 3.1.1. Expert identification and sampling strategy.

The research population was constructed using a purposive, non-probability sampling strategy designed to identify individuals with demonstrable expertise in AI-related fields. Rather than relying on self-declared labels, expertise was operationalized through a multi-criteria selection framework intended to maximize relevance, credibility, and reproducibility.

Candidate profiles were evaluated based on a combination of professional, academic, and activity-related indicators, including current employer, job title, and functional role, career trajectory, educational background, and sustained engagement on the platform. The primary sampling frame consisted of professionals affiliated with leading technology firms that play a significant role in AI research, development, deployment, or commercialization. These organizations included, but were not limited to, Alphabet (Google), Microsoft, Amazon, Meta, Apple, IBM, NVIDIA, Baidu, OpenAI, Alibaba Group, Anthropic, Tesla, Samsung Electronics, Intel, Oracle, SAP, Sony, Siemens, and xAI. In addition to industry practitioners, well-recognized AI researchers and thought leaders with strong academic or translational profiles were also included.

To further refine the definition of “expert,” additional screening criteria were applied. First, candidates were required to demonstrate a strong academic foundation in AI-relevant disciplines such as computer science, engineering, data science, or related technical and managerial fields, typically evidenced by degrees from internationally recognized universities. Second, professional or internship experience in innovation-driven technology organizations was prioritized to ensure practical exposure to advanced AI systems and workflows. Third, the sampling strategy intentionally covered a broad range of influential roles, including research, engineering, strategy, product management, operations, and executive leadership, in order to capture discourse spanning both technical and organizational dimensions of AI.

#### 3.1.2. Data collection procedure.

Data collection was conducted manually from publicly accessible LinkedIn profiles to ensure compliance with platform terms of service and ethical standards for social media research. No automated scraping tools or private data access mechanisms were used. For each selected profile, all publicly visible posts within the observation window were reviewed.

The data acquisition process took place over approximately two months and concluded in March 2025. The temporal coverage of the dataset spans posts published between 2015 and the date of collection. For each post, the full textual content and its corresponding timestamp were recorded. No personal identifiers beyond post-level metadata were retained.

#### 3.1.3. Dataset construction and filtering.

The dataset was constructed through a multi-stage filtering process. In the initial stage, several thousand LinkedIn profiles were screened, resulting in a preliminary cohort of 4,429 expert profiles. From this group, approximately 66,000 posts were collected.

In the second stage, a content-based filtering procedure was applied to isolate posts explicitly related to AI. This filtering step focused on AI-relevant terminology, concepts, and thematic relevance, removing posts unrelated to AI discourse (e.g., purely personal updates or non-technical professional content). After filtering, the final analytical corpus comprised approximately 22,000 AI-related posts authored by 2,692 unique experts.

#### 3.1.4. Data preparation and anonymization.

All posts in the final dataset were de-identified before analysis. Personal names, direct references to individuals, organizational promotions, URLs, emojis, and other potentially identifying elements were removed. The dataset retained only anonymized post identifiers, cleaned textual content, and temporal information necessary for analysis. Only English-language posts were included to ensure linguistic consistency in downstream NLP tasks.

The resulting dataset provides a large-scale, global, and professionally grounded corpus of expert discourse on AI, forming the empirical foundation for the topic modeling and analytical procedures described in the subsequent sections.

#### 3.1.5 Ethical considerations.

This study is based exclusively on publicly available LinkedIn posts collected manually in compliance with the platform’s terms of service. All personal identifiers were removed before analysis, and only de-identified post-level text was used. Given the public nature of the data and the anonymization procedures applied, formal ethical approval was not required under standard guidelines for social media research.

### 3.2 Data preprocessing and filtering

**Fig 3:**
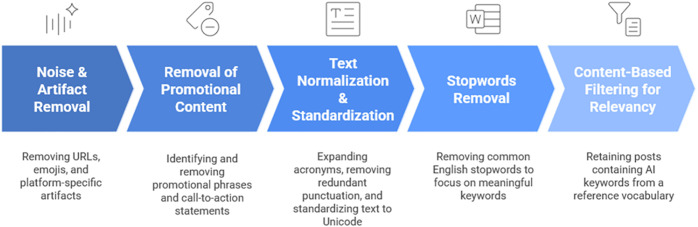
Data preprocessing and filtering.

The data preprocessing and filtering steps are presented in Fig 3.

To ensure the quality and validity of the analysis, a comprehensive data preprocessing and cleaning pipeline was implemented. This process, applied to the text of each post in the dataset, was designed to eliminate noise, standardize the text format, and filter for content relevant to the research questions. The pipeline consisted of the following sequential stages:

#### 3.2.1. Noise and artifact removal.

The initial step focused on removing extraneous elements that do not contribute semantic value to the text. This included removing all URLs, emojis, and residual HTML tags. Furthermore, platform-specific artifacts such as user mentions (e.g., @username) and hashtags (e.g., #keyword) were excised to maintain focus on the core content of the posts.

#### 3.2.2. Removal of promotional content.

Regular expressions (Regex) were employed to identify and eliminate promotional phrases and call-to-action statements (e.g., click here for more information). This step was crucial for purifying the dataset of commercial or persuasive language irrelevant to the thematic analysis.

#### 3.2.3. Text normalization and standardization.

To create a consistent and uniform text corpus, several normalization techniques were applied. First, common technical acronyms were expanded to their full form (e.g., AI was converted to artificial intelligence and ML to machine learning) to ensure semantic consistency. Second, all non-essential characters, such as redundant punctuation and excess whitespace, were removed. Finally, all text was standardized to the Unicode format to prevent encoding errors and ensure uniform character representation.

#### 3.2.4. Stopwords removal.

A standard list of English stopwords, common words with little semantic weight (e.g., and, the, is), was used to remove these high-frequency terms from the text. This step helps to focus the subsequent analysis on more meaningful, content-bearing keywords.

#### 3.2.5. Content-based filtering for relevancy.

Following the cleaning and normalization stages, a critical filtering process was conducted to isolate posts directly relevant to AI discussions. This was achieved by using a specialized reference vocabulary containing a set of keywords and phrases pertaining to AI and its sub-domains. A post was retained for the final dataset only if it contained at least one term from this reference vocabulary. This rigorous filtering ensured that the final corpus of approximately 22,000 posts was highly relevant and suitable for addressing the research questions.

This multi-stage preprocessing pipeline was instrumental in transforming the raw, noisy data into a clean, standardized, and highly relevant corpus, ready for the subsequent topic modeling analysis.

### 3.3. Topic modeling process

**Fig 4:**
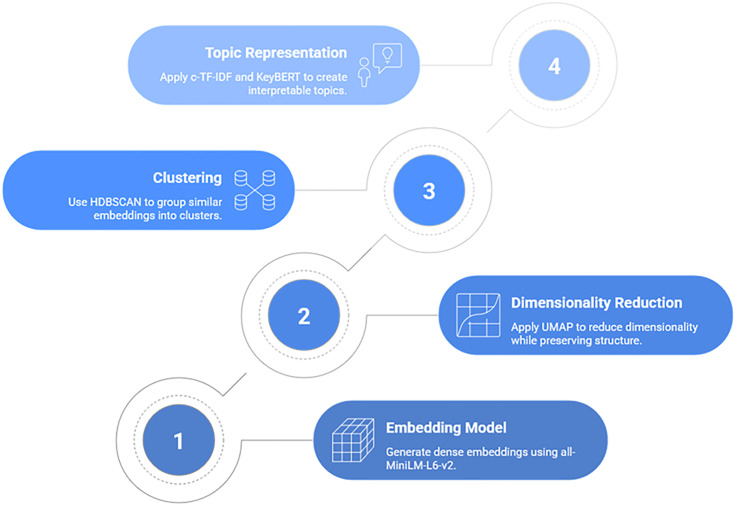
Topic modeling process.

The topic modeling process is presented in Fig 4.

To identify the underlying thematic structures within the dataset of experts’ posts, a topic modeling approach was employed. While traditional methods like Latent Dirichlet Allocation (LDA) [59] are commonly used, preliminary assessments indicated that LDA’s performance was unsatisfactory for this specific dataset. The short, informal, and noisy nature of social media text, combined with a wide and overlapping range of topics, limits the effectiveness of frequency-based models like LDA, which struggle to capture deeper semantic context.

To overcome these limitations, this study utilized BERTopic, an advanced, embedding-based topic modeling framework [60]. BERTopic leverages sentence-embedding models to capture the semantic meaning of text, followed by density-based clustering to form coherent topic clusters. This approach is significantly more effective for short and context-rich texts typical of social media.

The implementation of the BERTopic model was carefully configured with specific components and parameters to optimize performance for this dataset.

#### 3.3.1. Embedding model.

The all-MiniLM-L6-v2 model from the SentenceTransformer library was used to generate dense vector representations (embeddings) for each post [61,62].

#### 3.3.2. Dimensionality reduction.

The UMAP (Uniform Manifold Approximation and Projection) algorithm [63]was configured for dimensionality reduction with the following parameters: n_neighbors = 30, n_components = 5, and min_dist = 0.1, using cosine similarity as the distance metric.

#### 3.3.3. Clustering.

The HDBSCAN (Hierarchical Density-Based Spatial Clustering of Applications with Noise) algorithm was employed to cluster the reduced embeddings [64]. Key parameters were set to min_cluster_size = 15 and min_samples = 5.

#### 3.3.4. Topic representation.

For generating meaningful and interpretable topic representations, the class-based TF-IDF (c-TF-IDF) approach [60] was used via CountVectorizer. This was enhanced by removing a custom list of stopwords (e.g., intelligence, artificial, data, model) to prevent them from dominating the topic keywords. The KeyBERTInspired model was also used to further refine the quality of the topic labels.

Following the initial modeling process, the number of topics was programmatically reduced to a more consolidated and interpretable set of 13 distinct themes with topic_model.reduce_topics. The quality of this final model was then evaluated using the Coherence Score as a standard metric that measures the semantic similarity of the top words within a topic [65]. The final model achieved a Coherence Score of 0.4887.

While a coherence score of this value might appear moderate in studies using homogeneous corpora, it represents a suitable and reliable achievement given the unique challenges of the dataset. The LinkedIn data is not a uniform collection but a highly heterogeneous mix of personal posts, technical articles, informal comments, and diverse writing styles. This inherent diversity, coupled with a virtually unrestricted range of topics, presents a significant challenge for any topic modeling algorithm. Therefore, achieving this level of semantic coherence validates the effectiveness of the configured BERTopic model for extracting meaningful patterns from this complex, real-world data.

Following the initial topic modeling, the BERTopic framework produced a large set of preliminary topics, which were then programmatically consolidated into a smaller, interpretable set using the topic_model.reduce_topics method. The resulting clusters were iteratively examined to balance structural consistency with qualitative interpretability. Topic labels were first assigned by the lead researcher and subsequently reviewed and discussed by two additional researchers. Any disagreements were resolved through consensus, and the inherently interpretive nature of topic labeling is acknowledged as a limitation. Given the stochastic components involved in embedding-based clustering and dimensionality reduction, exact replication of document-to-topic assignments is not expected. Accordingly, the reported topics should be interpreted as indicative thematic structures that capture salient patterns in expert discourse, rather than as fixed or deterministic classifications.

## 4. Results

This section presents the findings from the topic modeling analysis of the 22,000 posts collected from technology experts. The results are structured to first provide a descriptive overview of the identified topics (answering RQ1: *What are the primary topics discussed by technology experts regarding Artificial Intelligence?*) and will subsequently be analyzed through the UTAUT framework in Section 5.2.

**Fig 5:**
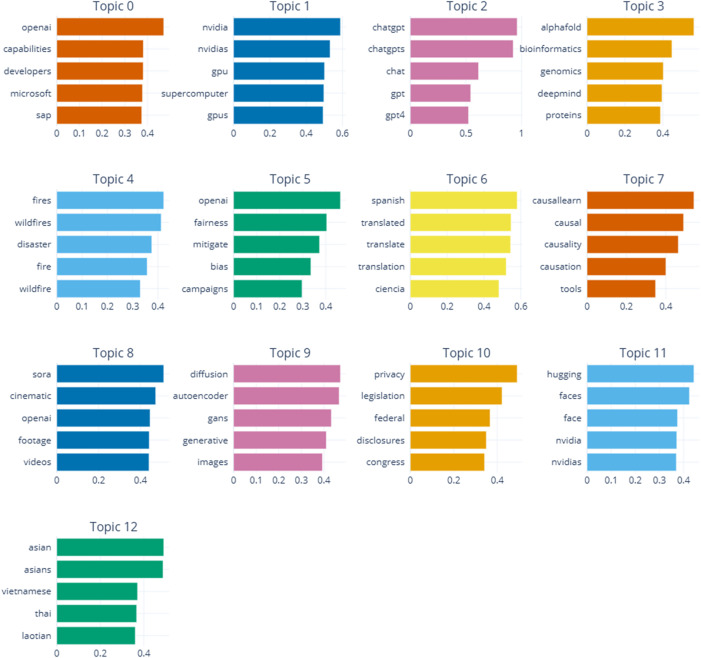
Top words per topic.

After a rigorous process of hyper-parameter tuning and model evaluation, a final model with 13 distinct topics was selected as the most suitable configuration. The number of topics provided an optimal balance between thematic comprehensiveness and differentiability, allowing for a feasible and meaningful interpretation of the results. The extracted topics cover various domains, including AI technologies, industrial applications, and ethical issues. Table 1. provides a summary of these topics, followed by a detailed interpretation of each. The top words identified for each topic are presented in Fig 5.

**Table 1:** Summary of topics.

Topic ID	Topic Label	No. of Posts	Top Keywords
0	Key Industry Players & Innovation	8,067	OpenAI, Microsoft, DeepMind, SAP, capabilities, developers, innovation, industry, cloud
1	AI Processing & Hardware	1,585	NVIDIA, GPUs, supercomputer, software, computing, training, GTC
2	Advanced Language Models (ChatGPT)	629	chatgpt, gpt, gpt4, app, ios, openai, code
3	AI in Biotechnology & Research	325	AlphaFold, bioinformatics, genomics, proteins, DeepMind, discovery, breakthrough
4	Natural Disaster Management	83	fires, wildfires, disaster, earthquake, flooded, imagery, insights
5	Ethics, Fairness, and Bias in AI	73	fairness, bias, mitigate, justice, integrity, media, tools
6	Language Translation (Spanish)	32	spanish, translated, translation, espanol, ciencia, aprendizaje
7	Causal Learning & Inference	29	causal, causality, causation, tools, knowledge, research, inference
8	AI in Video Generation (Sora)	26	sora, cinematic, footage, video, texttovideo, filmmakers
9	Generative Models for Image Synthesis	23	diffusion, autoencoder, gans, generative, images, texttoimage, denoising
10	Privacy Legislation & Regulation	16	privacy, legislation, federal, laws, congress, rulemaking
11	Face Recognition & Industry Hubs	15	hugging, faces, face, nvidia, hub, partnered, inferenceasaservice
12	Asian Communities & Regional Issues	15	asian, vietnamese, thai, indonesia, thailand, borneo, vietnam

### 4.1. Interpretation of identified topics

#### 4.1.1. Topic 0: key industry players & innovation.

Comprising 8,067 posts, this is the largest topic and centers on the key players and leading organizations in the AI domain. Keywords such as OpenAI, Microsoft, DeepMind, and SAP highlight the expert community’s focus on prominent industry actors. Furthermore, terms like capabilities, developers, innovation, industry, cloud, and development underscore the importance of technical capabilities, the central role of developers, and the foundational need for cloud infrastructure and continuous innovation. This topic reflects a forward-looking perspective on the opportunities presented by AI, emphasizing the pivotal role of major corporations in advancing the field.

#### 4.1.2. Topic 1: AI processing & hardware.

This topic, with 1,585 posts, focuses on the processing technologies and hardware infrastructure essential for AI. Keywords like NVIDIA, GPUs, and supercomputer point to the critical importance of powerful hardware for developing and deploying AI models. The inclusion of terms such as software, computing, and training indicates a focus on specialized software and the model training process. The keyword GTC, referring to NVIDIA’s annual conference, suggests that discussions also cover the latest industry news and innovations. This topic thus highlights the significance of hardware and software infrastructure in supporting AI.

#### 4.1.3. Topic 2: advanced language models (ChatGPT).

With 629 posts, this topic is specifically focused on advanced language models, particularly ChatGPT. Keywords such as chatgpt, gpt, and gpt4 demonstrate widespread expert attention on novel NLP technologies. terms like app and ios point to discussions around practical applications, especially in mobile environments. The presence of openai and code underscores the role of OpenAI and the software development aspects related to these models. Overall, this topic reflects the significant interest in and adoption of recent advancements in language models.

#### 4.1.4. Topic 3: AI in biotechnology & research.

This topic, containing 325 posts, is related to the application of AI in biotechnology and bioinformatics. Keywords like AlphaFold, bioinformatics, genomics, and proteins indicate a focus on AI’s advancements in analyzing biological data and protein structures. The terms DeepMind and discovery highlight the prominent role of companies like DeepMind in developing technologies such as AlphaFold, which have been instrumental in solving complex biomolecular problems and accelerating research. The presence of breakthroughs and researchers signifies the perception of these advancements as revolutionary steps in scientific research.

#### 4.1.5. Topic 4: natural disaster management.

Encompassing 83 posts, this topic primarily revolves around themes related to natural disasters and crisis management. Keywords such as fires, wildfires, and disaster show a specific focus on fire-related events. The presence of terms like buildings, earthquake, flooded, and imagery suggests discussions on structural damage and the use of satellite or aerial imagery for assessment and management of these crises. The keyword insights may indicate a focus on data analysis to provide better solutions for natural disaster response.

#### 4.1.6. Topic 5: ethics, fairness, and bias in AI.

This topic, with 73 posts, is centered on ethical issues and justice in the field of AI. Keywords such as fairness, bias, mitigate, and justice reveal the expert community’s focus on the challenges of discrimination and impartiality in AI development and application. Words like campaigns, media, and tools point to efforts to raise public awareness and use appropriate tools to combat bias. The keyword integrity emphasizes the importance of maintaining ethical soundness in the field.

#### 4.1.7. Topic 6: language translation.

Comprising 32 posts, this topic is mainly dedicated to discussions about translation and the Spanish language. keywords like spanish, translated, and espanol show a focus on the translation process. Additionally, the presence of ciencia (science) and aprendizaje (learning) suggests the importance of scientific and educational topics in this context.

#### 4.1.8. Topic 7: causal learning & inference.

This topic, with 29 posts, is centered on causal learning and cause-and-effect analysis. Keywords like causal, causality, and causation indicate a focus on understanding causal relationships in data. The terms tools, knowledge, research, and inference highlight the role of analytical tools and researchers in discovering and inferring these relationships, aiming to extract higher-quality knowledge from data.

#### 4.1.9. Topic 8: AI in video generation (Sora).

With 26 posts, this topic focuses on the generation and editing of video content using novel AI technologies. Keywords like sora, cinematic, footage, and texttovideo indicate a specific interest in generating cinematic videos from text prompts. The inclusion of openai, camera, and filmmakers highlights the role of advanced technologies in the filmmaking industry.

#### 4.1.10. Topic 9: generative models for image synthesis.

This topic of 23 posts is concentrated on generative models for digital image creation. Keywords such as diffusion, autoencoder, gans, and generative point to a focus on advanced techniques for content generation using deep learning. Terms like images, texttoimage, and denoising signify the application of these models in generating images from text and improving image quality.

#### 4.1.11. Topic 10: privacy legislation & regulation.

Comprising 16 posts, this topic is primarily dedicated to legal and regulatory issues related to privacy. Keywords like privacy, legislation, federal, and laws show a focus on governmental regulations concerning the protection of personal data. Words such as disclosures, congress, and rulemaking refer to legal processes and policy-making. This reflects expert concern for legal frameworks governing data security in the tech space.

#### 4.1.12. Topic 11: face recognition & industry hubs.

This topic, with 15 posts, focuses on technologies related to facial recognition and processing, as well as industry collaborations. The keyword hugging refers to the collaborative platform Hugging Face, while faces and face point to recognition technologies. The term nvidia highlights the company’s role in providing processing hardware. Keywords like hub and partnered refer to collaborative ecosystems, and terms such as inferenceasaservice signify the delivery of AI model-based services.

#### 4.1.13. Topic 12: asian communities & regional issues.

This topic, also with 15 posts, focuses on issues related to Asian communities. Keywords such as asian, vietnamese, thai, indonesia, and thailand indicate a focus on nationalities, cultures, and geographical regions within Asia, reflecting an awareness of regional and cultural matters within the broader technology discourse.

### 4.2. Topic analysis through the UTAUT framework

This section analyzes the 13 identified topics to address the second research question: *RQ2: How does an analysis of expert discourse inform, challenge, or re-contextualize the core constructs of the Unified Theory of Acceptance and Use of Technology (UTAUT) in the context of AI?* Each of the four core constructs of UTAUT will be examined in the subsequent subsections.

The topic modeling analysis revealed the natural structure of the expert discourse, characterized by a few high-frequency core topics and many of lower-frequency specialized topics. Confronted with this distribution, we made a deliberate methodological decision to retain and analyze all 13 identified topics, rather than discarding those with less frequent mentions. This decision was grounded in two principles. First, to maintain fidelity to the data, removing algorithmically-identified topics would risk an incomplete and biased representation of the discourse. Second, and more critically, the objective of this research extended beyond identifying dominant narratives to mapping the comprehensive, multifaceted landscape of the conversation around AI. The low-frequency topics, while smaller in volume, represent crucial niche domains, emerging trends, and specific concerns vital to certain segments of the expert community (e.g., AI in biotechnology, ethical considerations, causal learning). To ignore this “long tail” of the discourse would be to sacrifice analytical depth and richness for an oversimplified view of a complex phenomenon. Therefore, the inclusion of all topics was a conscious choice to capture both the breadth and depth of the expert discourse in its entirety.

To ensure a robust and theoretically grounded mapping, the authoring team engaged in a consensus-based mapping process. The topics were iteratively reviewed and discussed against the definitions of the UTAUT constructs until a unanimous agreement was achieved for all 13 topics.

#### 4.2.1. Performance expectancy.

PE, one of the most pivotal constructs in the UTAUT model, is fundamental to shaping individuals’ attitudes toward new technologies. As defined by Venkatesh et al. [8], it is the degree to which an individual believes that using the system will help them to attain gains in job performance. In most technology acceptance research, this belief in a technology’s utility and effectiveness is recognized as the strongest predictor of usage intention. Within the context of this study, which focuses on AI adoption by technology experts, PE reflects these professionals’ belief in AI’s potential to significantly enhance the quality and efficiency of their work, open up innovative pathways, accelerate scientific discovery, and ultimately boost their overall productivity and effectiveness.

The discourse among technology experts on LinkedIn clearly indicates that the belief in AI’s ability to drive fundamental transformations and deliver extensive performance benefits is a primary motivating force.

Foundational Enablers: Infrastructure and Industry Leaders: This performance expectation originates from observing the capabilities and innovations delivered by leading companies, supported by powerful infrastructure (Topics 0 and 1). The extensive discussion in **Topic 0 (Key Industry Players & Innovation)**, centered on major names like OpenAI, Microsoft, and DeepMind, and emphasizing concepts like capabilities and innovation, shows that experts view these organizations as flag bearers of AI’s high performance and the main drivers of transformation across the entire industry. This perception is reinforced by a deep understanding of **Topic 1 (AI Processing & Hardware)**, which highlights the critical role of powerful hardware from companies like NVIDIA and its GPUs in enabling the training of complex models and achieving new levels of computing. For these experts, infrastructural advancements are not merely technical improvements but gateways to unprecedented capabilities and innovations that directly translate to enhanced professional and industrial performance.

Tangible Applications and Generative AI: In the layer of more concrete applications, the emergence of advanced language models and generative AI capabilities (Topics 2, 8, 9, and 11) has revolutionized performance expectations in creativity, content production, and specialized services. The widespread discussion in **Topic 2 (Advanced Language Models)**, focusing on chatgpt and gpt4, signifies a profound belief in these models’ ability to solve problems, assist with code, and facilitate complex communication, directly leading to increased productivity in knowledge-based tasks. This expectation extends to visual content creation, as seen in the potential of tools like Sora in **Topic 8 (AI in Video Generation)** to produce cinematic content via texttovideo, and the ability of diffusion models and GANs in **Topic 9 (Generative Models for Images)** to create high-quality images from text. Furthermore, **Topic 11 (Face Recognition & Industry Hubs)**, with its focus on advanced facial recognition, inference-as-a-service offerings, and powerful models like Megatron, shows that experts expect AI to continuously provide new, high-performance functionalities for specific industrial and research applications.

Scientific Discovery and Complex Problem-Solving: PE is also deeply rooted in AI’s ability to advance scientific discovery and solve complex analytical problems (Topics 3, 7, and 4). Major successes like AlphaFold in bioinformatics and genomics (**Topic 3**), hailed as a breakthrough in accelerating scientific discovery, reinforce the belief that AI can deliver superhuman performance at the frontiers of knowledge. Similarly, the focus on **Topic 7 (Causal Learning)** and the use of inference tools for discovery of deeper relationships in data, as well as the application of AI to analyze imagery for insights in **Topic 4 (Natural Disaster Management)**, all point to an expectation that the technology will provide superior solutions and a deeper understanding that leads to better performance in critical decision-making and research.

Contextual Moderation of Performance Expectancy: Finally, the analysis reveals that the perception of PE can be moderated by contextual factors, as illustrated by **Topic 12 (Asian Communities & Regional Issues)**. While a system may exhibit high technical performance globally (as discussed in Topics 0 or 2), it may not meet the performance expectations of experts focused on the Asian market if it fails to address specific linguistic needs, cultural nuances, or infrastructural challenges. For these experts, a system’s performance is judged not only by its general technical capabilities but also by its adaptability and effectiveness within a specific regional and cultural context. This indicates that performance is a nuanced concept for experts, deeply intertwined with the context of application and the potential for tangible value creation.

In conclusion, this study reveals that PE for technology experts is not a monolithic construct of perceived utility, but a multi-layered framework. It is built upon a foundation of infrastructural power (Topic 0, 1), actualized through tangible generative applications (Topics 2, 8, 9, 11), and validated by its capacity to drive frontier-level scientific discovery (Topics 3, 4, 7). Crucially, the perceived value of these layers is not absolute but is contextually moderated by specific regional and cultural demands (Topic 12). This re-contextualization suggests that for expert users, the ‘performance’ of a technology is inseparable from its ecosystem, its innovative trajectory, and its adaptability, challenging a simpler, task-oriented view of the construct.

This multi-layered evidence suggests that for an expert population, the ‘performance’ construct is re-contextualized, moving beyond individual job performance to encompass industry-wide transformative potential and frontier-level scientific discovery.

#### 4.2.2. Effort expectancy.

EE, the second key construct in the UTAUT model, pertains to the degree of ease associated with the use of the system [8]. This construct represents the user’s perception of the cognitive and operational effort required to learn, use, and effectively interact with a new technology. Although EE often has a more pronounced impact in the early stages of adoption or for users with lower technical proficiency, it remains a relevant factor even for experts. Excessive complexity or poor usability can act as a deterrent, or at least affect the speed and scope of adoption. For technology experts dealing with advanced AI platforms, EE relates not only to the ease of use of a specific tool but also to broader aspects such as system transparency, integration with existing workflows, and the continuous learning required to keep pace with changes.

The discourse among these experts reveals that while their high technical skills may increase their tolerance for complexity, EE is still a significant factor in their evaluation of AI technologies, particularly for tools that require frequent, direct interaction or that aim to simplify complex processes.

User-Centric Design and Accessibility: The discussions surrounding **Topic 2 (Advanced Language Models)** show that the appeal of models like ChatGPT stems not only from their functional capabilities but also from their accessible delivery through familiar interfaces such as mobile apps and on iOS. This highlights the importance of user-friendly design principles even for experts; they expect intuitive interfaces, short learning curves, and the ability to efficiently use and apply complex AI tools. Furthermore, references to code in this context can be interpreted as an expectation for well-structured (Application Programming Interface) APIs and clear documentation, which reduces the development effort required to integrate these models into new applications. This demand for clarity also hints at an underlying need for system transparency, where experts expect to understand how a model can be reliably controlled and integrated, not just use it as a ‘black box’.

Simplification of Complex and Creative Workflows: A key aspect of EE for experts is the potential of AI to reduce the cognitive load and simplify previously laborious tasks. **Topic 8 (AI in Video Generation)**, with its focus on text-to-video capabilities, exemplifies how AI can streamline creative and technical processes that once required significant time and specialized skills. Such tools lower the technical barrier for filmmakers and a broader range of professionals who need to produce high-quality visual content with substantially less effort. Similarly, **Topic 6 (Language Translation)**, with its focus on translation and aprendizaje (learning), illuminates how AI tools can reduce the mental effort required to access and understand international information, thereby facilitating knowledge acquisition.

The Moderating Role of Cultural and Linguistic Context: The perception of EE is also significantly moderated by cultural and linguistic context, as suggested by the discussions in **Topic 12 (Asian Communities)**. AI tools predominantly designed with Western users and the English language in mind may require substantially more effort from experts in Asian countries to adapt, learn, and use effectively. This added effort can arise from language barriers in user interfaces or documentation, or from cultural mismatches in design paradigms and visual cues. An interface that is intuitive in one culture may require more learning in another. Therefore, for experts working in or designing solutions for these contexts, EE is heavily influenced by the degree of localization and adaptation to specific cultural and linguistic needs.

In summary, the evidence reveals that for technology experts, EE transcends its traditional definition of basic usability. It is re-contextualized as an expectation for cognitive and operational efficiency. This is manifested in the demand for frictionless access and integration through intuitive interfaces and well-documented APIs (Topic 2), and the desire for AI to act as a simplification engine that reduces the cognitive load of complex creative and analytical tasks (Topics 6, 8). Crucially, the perception of this efficiency is not universal; it is significantly moderated by the cultural and linguistic context, where a lack of localization can impose a substantial effort burden (Topic 12). Therefore, for experts, low effort is not about overcoming a lack of skill, but about maximizing their own productivity by leveraging tools that are seamlessly integrated, conceptually simple, and contextually intelligent.

Thus, for experts, EE is not about overcoming a lack of technical skill, but about maximizing cognitive efficiency through seamless integration and simplification of complex tasks, a more sophisticated interpretation of the original construct.

#### 4.2.3. Social influence.

SI, the third core construct in the UTAUT model, refers to the degree to which an individual perceives that those important others believe they should use a new system. While this is true for any user, for the technology experts in this study, SI operates through a unique, dual mechanism. They are not merely passive **receivers** of influence from peers, industry leaders, and scientific breakthroughs. As key nodes in the innovation network, they also act as powerful **shapers** and amplifiers of these very same influences, creating a recursive loop of norm formation. This section explores this dynamic, examining how experts are influenced by dominant industry and scientific trends (Topics 0, 1, 3, 11), while also engaging with and shaping broader societal and regulatory pressures (Topics 5, 10).

Influence from Industry Leaders and Scientific Validation: In **Topic 0 (Key Industry Players & Innovation)**, the activities and announcements from companies like OpenAI, Microsoft, and DeepMind are followed not just for their technological output but as trend-setters that confer social legitimacy upon new technologies. By introducing industry-standard platforms, they implicitly signal to experts that adopting these tools is desirable professional practice. This effect is reinforced by the focus on NVIDIA and its influential GTC conference in **Topic 1 (AI Processing & Hardware)**, and by the introduction of new technology launches and collaborative platforms like the Hugging Face Hub in **Topic 11 (Face Recognition & Industry Hubs)**, all of which shape norms around accepted tools within the expert community. Scientific success plays a similar role; as seen in **Topic 3 (AI in Biotechnology)**, breakthroughs like AlphaFold by DeepMind dramatically increase AI’s credibility among researchers, encouraging others to adopt and explore these technologies based on evidence validated by the scientific community.

Broader Societal and Regulatory Pressures: At another level, wider social discourses regarding the ethical and legal implications of AI (Topics 5 and 10) increasingly exert influence on technology experts. **Topic 5 (Ethics, Fairness, and Bias in AI)**, with its focus on fairness, bias, and justice, reveals experts’ engagement with these critical challenges. These discussions, often amplified by the media and awareness campaigns, create significant social pressure to develop and deploy AI responsibly. Similarly, **Topic 10 (Privacy Legislation & Regulation)**, with its focus on privacy and legislation, shows that legal frameworks and public concerns about data protection act as a social force influencing how experts approach the use and development of AI systems. These factors shape the social climate around AI and can act as drivers for, or barriers against, the adoption of certain applications.

The Moderating Role of Regional and Cultural Norms: The influence of regional context, as highlighted by **Topic 12 (Asian Communities)**, can further moderate how experts perceive and react to SI. While a technology might be heavily promoted by global thought leaders (Topic 0), its adoption in a specific Asian country could be more strongly influenced by local cultural, social, or political norms. Concerns related to privacy (Topic 10), for example, might be interpreted differently based on local laws and cultural values, leading to different social pressures. This suggests that for experts operating within or targeting these regions, the important others and reference norms can differ significantly from global trends, thus modulating their response to broader SI.

In conclusion, SI for technology experts is not a one-way street but a dynamic, multi-layered feedback loop. While they are influenced by the legitimacy conferred by industry leaders and the validation from the scientific community, their active engagement, adoption, and discourse simultaneously reinforce and shape these very norms for the wider professional community. Their attention is therefore twofold: they monitor what is professionally and ethically acceptable, while simultaneously defining it for others. This dual role as both the audience and the authors of social norms, further complicated by regional and cultural variations, presents a more complex and recursive model of SI than is typically considered in UTAUT studies.

This reveals a more complex, recursive model of SI than typically considered, where experts are simultaneously the audience for and the authors of professional and ethical norms, expanding the construct beyond simple peer compliance.

#### 4.2.4. Facilitating conditions.

FCs, the fourth foundational construct in the UTAUT model, refers to the degree to which an individual believes that an organizational and technical infrastructure exists to support use of the system [8]. This construct encompasses the tangible resources and support available to a user, which can include hardware, software, technical expertise, training, and financial resources. Unlike the other three constructs that primarily influence behavioral intentions, FCs often have a more direct impact on actual usage behavior, as it reflects the practical enablers and barriers an individual faces. For the technology experts in this study, access to advanced and appropriate FCs is not just an advantage but a necessity for research, development, and implementation of innovative AI solutions.

The expert discourse clearly reveals the breadth of FCs required to advance the field of AI, extending beyond technical tools to include human and knowledge ecosystems.

***Foundational Technical Infrastructure:*** At the forefront are the foundational technological infrastructures and advanced platforms. As observed in **Topic 0 (Key Industry Players & Innovation)**, with its references to cloud services and tools for developers, and **Topic 1 (AI Processing & Hardware)**, with its emphasis on NVIDIA, GPUs, and supercomputers, it is clear that cloud platforms and specialized high-performance hardware form the backbone of most AI activities. Furthermore, **Topic 11 (Face Recognition & Industry Hubs)** highlights the role of collaborative platforms like the Hugging Face Hub and new service models like inference-as-a-service as key technical enablers.

***Access to Specialized Tools, APIs, and Data:*** Beyond macro-infrastructure, access to specific software tools, APIs, and relevant data is another critical facilitating condition. In **Topic 2 (Advanced Language Models)**, references to code and APIs from organizations like OpenAI signify the importance of these interfaces in enabling experts to integrate advanced model capabilities into their own systems. Similarly, **Topic 7 (Causal Learning)** and **Topic 9 (Generative Models for Images)** both emphasize the importance of specialized software tools and modeling techniques as enablers of research and innovation. The need for access to relevant data, such as imagery in **Topic 4 (Natural Disaster Management)**, is also highlighted as a crucial facilitator for critical AI applications.

***Human Capital and Knowledge Resources:*** FCs are not limited to technical infrastructure; skilled human resources and continuous learning opportunities are equally vital. The prominent mention of developers (Topic 0), researchers (Topics 3 and 7), and filmmakers (Topic 8) indicates that the availability of this talent is itself a facilitating condition for projects and companies. Furthermore, discussions around model training (Topic 1) and aprendizaje (learning, Topic 6) can be interpreted more broadly as the importance of training programs, quality documentation, and knowledge-sharing platforms for upskilling both experts and the next generation of AI professionals.

***Ecosystem and Regulatory Frameworks:*** Finally, the broader ecosystem provides FCs. By highlighting tools to mitigate bias, **Topic 5 (Ethics, Fairness, and Bias in AI)** suggests that the availability of such methods facilitates responsible AI development. Concurrently, **Topic 10 (Privacy Legislation & Regulation)** indicates that a transparent and supportive legal environment (legislation, laws) can reduce uncertainty and act as a significant facilitator for investment and innovation in AI.

***The Moderating Role of Regional Context:*** The perception of these conditions is moderated by regional context, as seen in **Topic 12 (Asian Communities)**. While access to advanced cloud platforms (Topic 0) is a general facilitator, its quality, accessibility, or need for localization may differ in Asian regions. Similarly, the availability of local talent, culturally adapted training programs, and supportive national policies are all specific FCs that moderate the sufficiency of global resources.

In conclusion, the expert discourse radically expands the traditional scope of FCs. It moves beyond a simple inventory of technical resources to a holistic ecosystem perspective, where organizational and technical infrastructure are deeply intertwined with human capital and supportive regulatory frameworks. For experts, the existence of powerful hardware and APIs is a necessary but insufficient condition. True facilitation arises from the combined effect of these tools with available talent, knowledge-sharing platforms, and clear ethical and legal guidelines that reduce uncertainty and foster responsible innovation. This ecosystem view, whose adequacy is itself moderated by regional contexts, suggests that to support expert-level AI adoption, a fragmented approach is ineffective. Instead, a comprehensive strategy that nurtures technology, talent, and transparent governance in tandem is essential.

This holistic ecosystem perspective, encompassing technology, talent, and governance, radically expands the traditional scope of FCs, suggesting that for expert-level AI adoption, a fragmented inventory of resources is insufficient.

**Fig 6:**
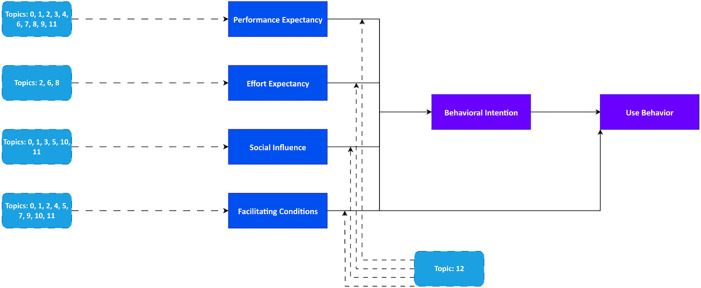
Integrating topic modeling insights with UTAUT to investigate AI adoption among experts.

The analysis of the thirteen topics within the framework of the UTAUT constructs has led to the formation and clarification of a conceptual model for understanding AI adoption among technology experts. In this resulting model, each of the extracted topics serves as empirical evidence, illustrating how the constructs of PE, EE, SI, and FCs are manifested in the discourse of this expert group. Furthermore, the moderating effect of cultural context (as represented by Topic 12) on these relationships has been integrated as a key component of the model. The structure of this integrative framework is illustrated in Fig 6, which visually represents the alignment between the identified topics and the core UTAUT constructs, as well as the moderating role of cultural context in shaping these relationships.

## 5. Discussion

This study sought to identify the primary topics of expert discourse surrounding AI and to understand these themes through the lens of the UTAUT. The topic modeling analysis revealed 13 distinct topics, ranging from key industry players and hardware infrastructure (Topics 0, 1) to specific generative AI applications (Topics 2, 8, 9), and broader societal concerns such as ethics and regulation (Topics 5, 10). The subsequent analysis demonstrated that these topics map coherently onto the four core constructs of UTAUT, providing a nuanced model of AI acceptance from the expert perspective. This final section discusses the theoretical and practical implications of these findings, as well as the study’s limitations and avenues for future research.

### 5.1. Theoretical implications

This research contributes to the technology acceptance literature by directly addressing how the core constructs of UTAUT should be re-contextualized for a population of technology experts. Our analysis of the expert discourse provides empirical evidence for the following four key theoretical implications:

1) For this expert community, PE transcends simple task efficiency. It is redefined by the potential for major scientific breakthroughs and industry-wide innovation, as evidenced by the intense focus on transformative achievements like AlphaFold (Topic 3) and the innovations driven by key industry players (Topic 0).
2) EE is not about overcoming basic usability issues, but about maximizing cognitive efficiency. This is demonstrated by the demand for seamless integration through well-documented APIs (Topic 2) and tools that dramatically simplify complex creative workflows, such as text-to-video generators (Topic 8).
3) SI evolves from simple peer pressure into a dynamic, dual role where experts are both receivers and shapers of norms. This is shown by their simultaneous attention to trends set by industry leaders (Topic 0) and their engagement with broader societal pressures regarding ethics and bias (Topic 5).
4) FCs are viewed not as a simple checklist of tools, but as a holistic ecosystem. True facilitation arises from the interaction between powerful technical infrastructure (Topic 1), collaborative knowledge hubs (Topic 11), and supportive governance frameworks (Topic 10), moving far beyond the traditional definition of the construct.

Our findings highlight the critical moderating role of cultural and regional context. The analysis of Topic 12 (Asian Communities) highlighted meaningful contextual variation in expert discourse, indicating that the interpretation of UTAUT constructs is shaped by local needs, norms, and infrastructure.

**Fig 7:**
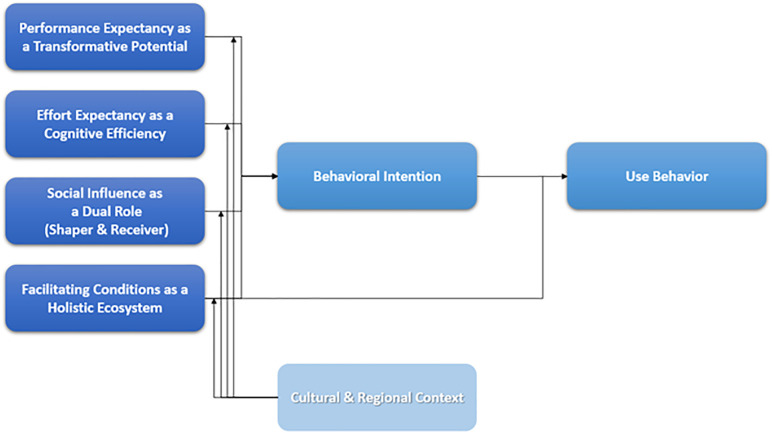
The Re-contextualized UTAUT: A Model of AI Acceptance by Experts. This is a conceptual model derived from the qualitative analysis of expert discourse. The arrows represent hypothesized relationships based on the extended UTAUT framework, which can be empirically tested in future quantitative research.

This finding supports the need to re-contextualize UTAUT beyond a one-size-fits-all model in global AI adoption research. Finally, this study demonstrates the value of a novel mixed-methodological approach—combining computational topic modeling with established theoretical frameworks—to analyze large-scale, real-world discourse. This method provides a powerful way to generate empirical, data-driven insights into the complex dynamics of technology acceptance. The resulting re-contextualized UTAUT model of AI acceptance among experts is presented in Fig 7.

### 5.2. Practical and managerial implications

The findings of this study offer several actionable insights for various stakeholders:

#### 5.2.1. For technology companies and marketers.

The dominance of PE in the expert discourse suggests that marketing should transcend generic claims and feature lists, and instead showcase evidence of transformative impact. Our findings indicate this influential community is less swayed by simple product announcements and more by demonstrable, AlphaFold-level breakthroughs and access to powerful, open ecosystems (e.g., robust APIs and collaborative platforms like Hugging Face), which should become the core of strategic communication.

#### 5.2.2. For R&D and product managers.

The detailed manifestations of EE and FCs underscore that even for experts, a seamless developer experience is crucial. This includes providing well-documented APIs, robust cloud support, and fostering collaborative platforms (e.g., Hugging Face) to lower barriers to adoption and experimentation.

#### 5.2.3. For policymakers and ethicists.

The organic emergence of dedicated topics on ethics, bias, and privacy regulation (Topics 5 and 10) confirms that these are not fringe issues but core components of the expert conversation. This provides a strong mandate for policymakers to engage directly with the expert community in developing timely and effective governance frameworks.

#### 5.2.4. For investors and strategists.

The identified topics serve as a leading indicator of technological trends and areas of high interest within the expert community. The focus on generative AI for various modalities (text, images, video), specialized hardware, and applications in fields like biotechnology can help guide strategic investment and resource allocation.

### 5.3. Limitations and future research

This study has several limitations that offer avenues for future research. First, the data is sourced exclusively from LinkedIn, and the discourse may differ on other platforms like X (formerly Twitter), academic forums, or private channels. Future studies could conduct comparative analyses across platforms. Second, our definition of expert, while systematically applied, is one of many possible operationalizations. Alternative criteria could yield different results. Third, the study is descriptive and exploratory; it identifies and analyzes themes but does not make causal claims about adoption behavior.

In addition, several contextual considerations should be acknowledged. First, the dataset consists primarily of posts written in English, which may underrepresent perspectives from non-English-speaking experts and limit the generalizability of observed trends across global professional communities. Second, our focus on leading technology firms and prominent AI researchers may introduce a bias toward viewpoints prevalent in large organizations, potentially underrepresenting smaller companies, startups, or independent practitioners. Finally, while the analysis captures patterns and thematic structures in professional discussions, it does not directly measure actual AI adoption behaviors. Accordingly, the findings should be interpreted as indicative of expert discourse.

Future research could build on these findings by:

1. Employing surveys or interviews to quantitatively test the relationships inferred in our model.
2. Conducting a longitudinal study to track the evolution of these topics over a longer timeframe.
3. Applying this mixed-method approach to expert discourse in other languages or regions to further explore the role of cultural context.
4. Incorporating the UTAUT moderators (e.g., age, experience), if a dataset with such demographic information becomes available.

### 5.4. Conclusion

In conclusion, by systematically analyzing the discourse of thousands of technology experts, this study moves beyond a simple application of UTAUT to propose a re-contextualized model of AI acceptance for its most critical adopters. The findings reveal that for this expert community, adoption is not a simple calculation but a complex negotiation. It is driven by an expectation of transformative performance, contingent upon a holistic ecosystem of facilitators, and weighed against the demand for cognitive efficiency [66,67]. Crucially, this process is framed by their dual role in Social Influence, where they not only react to norms but actively shape the social, ethical, and professional standards of their field. Therefore, grasping this nuanced expert perspective provides a tangible strategic advantage for anyone seeking to navigate and lead in the AI era.

## References

[1] Holzinger A, Kieseberg P, Weippl E, Tjoa AM. Current advances, trends and challenges of machine learning and knowledge extraction: From machine learning to explainable AI. Machine Learning and Knowledge Extraction. 2018.

[2] Sami M. Foundations of Artificial Intelligence and Applications. JAIT. 2022;2(1):1–2. doi: 10.37965/jait.2022.01

[3] Volmer J, Sonnentag S. The role of star performers in software design teams. Journal of Managerial Psychology. 2011;26(3):219–34. doi: 10.1108/02683941111112659

[4] Wang L, Ye K, Liu Y, Wang W. Factors affecting expert performance in bid evaluation: An integrated approach. Frontiers in Psychology. 2022;13. doi: 10.3389/fpsyg.2022.819692 PMC9387678 35992487

[5] Sauer CM, Skaik S, Tumpa RJ. Architects and designers on LinkedIn: perceptions and strategies for professional success. Engineering, Construction and Architectural Management. 2024.

[6] Farzindar A, Inkpen D, Hirst G. Natural language processing for social media. Springer. 2015.

[7] V R, Kodmelwar MK, S AJ, Shekhawat J, Sakhare NN, Vijayalakshmi VJ. Retracted: Unlocking the Power of Natural Language Processing to Automate Knowledge Discovery. In: 2023 3rd International Conference on Smart Generation Computing, Communication and Networking (SMART GENCON), 2023. 1–6. doi: 10.1109/smartgencon60755.2023.10442434

[8] Venkatesh V, Morris MG, Davis GB, Davis FD. User Acceptance of Information Technology: Toward A Unified View1. MIS Quarterly. 2003;27(3):425–78. doi: 10.2307/30036540

[9] Radhakrishnan J, Chattopadhyay M. Determinants and Barriers of Artificial Intelligence Adoption – A Literature Review. IFIP Advances in Information and Communication Technology. Springer International Publishing. 2020:89–99. doi: 10.1007/978-3-030-64849-7_9

[10] Pathak A, Bansal V. Factors influencing the readiness for artificial intelligence adoption in Indian insurance organizations. In: International Working Conference on Transfer and Diffusion of IT, 2024.

[11] Horani OM, Al-Adwan AS, Yaseen H, Hmoud H, Al-Rahmi WM, Alkhalifah A. The critical determinants impacting artificial intelligence adoption at the organizational level. Information Development. 2023;41(3):1055–79. doi: 10.1177/02666669231166889

[12] Khanfar AA, Kiani Mavi R, Iranmanesh M, Gengatharen D. (2025). Factors influencing the adoption of artificial intelligence systems: a systematic literature review. Management Decision.

[13] Siddiqui HA, Khan A, Shaikh S. Unveiling Barriers and Enablers: A Study on AI Adoption in Business Management. jsom. 2025;4(1):193–209. doi: 10.56976/jsom.v4i1.179

[14] Hoffman J, Wenke R, Angus RL, Shinners L, Richards B, Hattingh L. Overcoming barriers and enabling artificial intelligence adoption in allied health clinical practice: A qualitative study. Digit Health. 2025;11. doi: 10.1177/20552076241311144 39906878 PMC11792011

[15] Mkhize T, Oosterwyk G, Kautondokwa P. Examining the barriers and enablers of AI adoption in a multimedia organization. In: Proceedings of Society, 2023. 117–28.

[16] Madanchian M, Taherdoost H. Barriers and Enablers of AI Adoption in Human Resource Management: A Critical Analysis of Organizational and Technological Factors. Information. 2025;16(1):51. doi: 10.3390/info16010051

[17] Bedué P, Fritzsche A. Can we trust AI? An empirical investigation of trust requirements and guide to successful AI adoption. JEIM. 2021;35(2):530–49. doi: 10.1108/jeim-06-2020-0233

[18] Lingling M. Do Bulgarian SMEs differ from their international peers in adoption of AI? In: Proceedings of the International Conference on Business Excellence, 2025.

[19] Brey B, van der Marel E. The role of human-capital in artificial intelligence adoption. Economics Letters. 2024;244:111949. doi: 10.1016/j.econlet.2024.111949

[20] Dahlke J, Beck M, Kinne J, Lenz D, Dehghan R, Wörter M, et al. Epidemic effects in the diffusion of emerging digital technologies: evidence from artificial intelligence adoption. Research Policy. 2024;53(2):104917. doi: 10.1016/j.respol.2023.104917

[21] Barnes AJ, Zhang Y, Valenzuela A. AI and culture: Culturally dependent responses to AI systems. Current Opinion in Psychology. 2024;101838. 39002473 10.1016/j.copsyc.2024.101838

[22] Frimpong V. Cultural and Regional Influences on Global AI Apprehension. 2024.

[23] Aruna K, Kumari YS, Parla S, Naveen S, Galavilli S, Raj A. The social impact of emerging technologies: a comparative study of ai adoption across cultures. ShodhKosh J Vis Per Arts. 2024;5(4). doi: 10.29121/shodhkosh.v5.i4.2024.2599

[24] Tubadji A, Denney T, Webber DJ. Cultural relativity in consumers’ rates of adoption of artificial intelligence. Economic Inquiry. 2021;59(3):1234–51. doi: 10.1111/ecin.12978

[25] Murire OT. Artificial intelligence and its role in shaping organizational work practices and culture. Administrative Sciences. 2024;14(12):316.

[26] Davis FD. Perceived Usefulness, Perceived Ease of Use, and User Acceptance of Information Technology. MIS Quarterly. 1989;13(3):319–40. doi: 10.2307/249008

[27] Rogers EM, Singhal A, Quinlan MM. Diffusion of innovations. An integrated approach to communication theory and research. Routledge. 2014:432–48.

[28] Fishbein M, Ajzen I. Belief, attitude, intention, and behavior: An introduction to theory and research. 1977.

[29] Ajzen I. The theory of planned behavior. Organizational Behavior and Human Decision Processes. 1991;50(2):179–211. doi: 10.1016/0749-5978(91)90020-t

[30] Andrews JE, Ward H, Yoon J. UTAUT as a Model for Understanding Intention to Adopt AI and Related Technologies among Librarians. The Journal of Academic Librarianship. 2021;47(6):102437. doi: 10.1016/j.acalib.2021.102437

[31] Das S, Datta B. Application of UTAUT2 on Adopting Artificial Intelligence Powered Lead Management System (AI-LMS) in passenger car sales. Technological Forecasting and Social Change. 2024;201:123241. doi: 10.1016/j.techfore.2024.123241

[32] Hmoud BI, Várallyai L. Artificial Intelligence in Human Resources Information Systems: Investigating its Trust and Adoption Determinants. IJEMS. 2020;5(1):749–65. doi: 10.21791/ijems.2020.1.65

[33] Kelly S, Kaye S-A, Oviedo-Trespalacios O. What factors contribute to the acceptance of artificial intelligence? A systematic review. Telematics and Informatics. 2023;77:101925. doi: 10.1016/j.tele.2022.101925

[34] Hameed BZ, Naik N, Ibrahim S, Tatkar NS, Shah MJ, Prasad D, et al. Breaking Barriers: Unveiling Factors Influencing the Adoption of Artificial Intelligence by Healthcare Providers. BDCC. 2023;7(2):105. doi: 10.3390/bdcc7020105

[35] Yunji C, Qi G. AI for technology: Applied practices and future perspectives of technological intelligence in high tech areas. Bulletin of Chinese Academy of Sciences. 2024;39(1):34–40. doi: 10.16418/j.issn.1000-3045.20231123004

[36] Sari PK, Ramadan F, Murti YR. Examining Students’ Motivation to Continue Using AI-Chatbot for Academic Assignment. J Sistem Inf (J Inf Sys). 2024;20(2):18–31. doi: 10.21609/jsi.v20i2.1417

[37] Hong J. Artificial intelligence (AI), don’t surprise me and stay in your lane: An experimental testing of perceiving humanlike performances of AI. Human Behav and Emerg Tech. 2021;3(5):1023–32. doi: 10.1002/hbe2.292

[38] Gökçe Tekin Ö. Factors affecting teachers’ acceptance of artificial intelligence technologies: analyzing teacher perspectives with structural equation modeling. ITALL. 2024. doi: 10.52911/itall.1532218

[39] Tran AQ, Nguyen LH, Nguyen HSA, Nguyen CT, Vu LG, Zhang M, et al. Determinants of Intention to Use Artificial Intelligence-Based Diagnosis Support System Among Prospective Physicians. Front Public Health. 2021;9:755644. doi: 10.3389/fpubh.2021.755644 34900904 PMC8661093

[40] Tanantong T, Wongras P. A UTAUT-Based Framework for Analyzing Users’ Intention to Adopt Artificial Intelligence in Human Resource Recruitment: A Case Study of Thailand. Systems. 2024;12(1):28. doi: 10.3390/systems12010028

[41] Terblanche N, Cilliers D. Factors that influence users’ adoption of being coached by an artificial intelligence coach. Philosophy of Coaching: An International Journal. 2020;5(1):61–70.

[42] Méndez-Suárez M, Monfort A, Hervas-Oliver J-L. Are you adopting artificial intelligence products? Social-demographic factors to explain customer acceptance. European Research on Management and Business Economics. 2023;29(3):100223. doi: 10.1016/j.iedeen.2023.100223

[43] Belanche D, Casaló LV, Flavián C. Artificial Intelligence in FinTech: understanding robo-advisors adoption among customers. IMDS. 2019;119(7):1411–30. doi: 10.1108/imds-08-2018-0368

[44] Alanzi T, Almahdi R, Alghanim D, Almusmili L, Saleh A, Alanazi S, et al. Factors Affecting the Adoption of Artificial Intelligence-Enabled Virtual Assistants for Leukemia Self-Management. Cureus. 2023;15(11):e49724. doi: 10.7759/cureus.49724 38161825 PMC10757561

[45] Cheng M, Li X, Xu J. Promoting Healthcare Workers’ Adoption Intention of Artificial-Intelligence-Assisted Diagnosis and Treatment: The Chain Mediation of Social Influence and Human-Computer Trust. Int J Environ Res Public Health. 2022;19(20):13311. doi: 10.3390/ijerph192013311 36293889 PMC9602845

[46] Dabbous A, Aoun Barakat K, Merhej Sayegh M. Enabling organizational use of artificial intelligence: an employee perspective. JABS. 2021;16(2):245–66. doi: 10.1108/jabs-09-2020-0372

[47] Flathmann C, Duan W, Mcneese NJ, Hauptman A, Zhang R. Empirically Understanding the Potential Impacts and Process of Social Influence in Human-AI Teams. Proc ACM Hum-Comput Interact. 2024;8(CSCW1):1–32. doi: 10.1145/363732639286336

[48] Khanijahani A, Iezadi S, Dudley S, Goettler M, Kroetsch P, Wise J. Organizational, professional, and patient characteristics associated with artificial intelligence adoption in healthcare: A systematic review. Health Policy and Technology. 2022;11(1):100602. doi: 10.1016/j.hlpt.2022.100602

[49] Pai V, Chandra S. Exploring Factors Influencing Organizational Adoption of Artificial Intelligence (AI) in Corporate Social Responsibility (CSR) Initiatives. PAJAIS. 2022;14:82–115. doi: 10.17705/1pais.14504

[50] Gallivan MJ, Spitler VK, Koufaris M. Does Information Technology Training Really Matter? A Social Information Processing Analysis of Coworkers’ Influence on IT Usage in the Workplace. Journal of Management Information Systems. 2005;22(1):153–92. doi: 10.1080/07421222.2003.11045830

[51] Sood A, Bhardwaj AK, Sharma RK. Perceptions of facilitators towards adoption of AI-based solutions for sustainable agriculture. Journal of Decision Systems. 2023;:1–35. doi: 10.1080/12460125.2023.2294398

[52] Jarvie-Eggart M, Owusu-Ansah A, Stockero S. Facilitating Conditions for Engineering Faculty Technology Adoption. In: 2022 ASEE Annual Conference & Exposition Proceedings. doi: 10.18260/1-2--41942

[53] Grover P, Kar AK, Dwivedi YK. Understanding artificial intelligence adoption in operations management: insights from the review of academic literature and social media discussions. Ann Oper Res. 2020;308(1–2):177–213. doi: 10.1007/s10479-020-03683-9

[54] Solaimani S, Swaak L. Critical Success Factors in a multi-stage adoption of Artificial Intelligence: A Necessary Condition Analysis. Journal of Engineering and Technology Management. 2023;69:101760. doi: 10.1016/j.jengtecman.2023.101760

[55] Strohm L, Hehakaya C, Ranschaert ER, Boon WPC, Moors EHM. Implementation of artificial intelligence (AI) applications in radiology: hindering and facilitating factors. Eur Radiol. 2020;30(10):5525–32. doi: 10.1007/s00330-020-06946-y 32458173 PMC7476917

[56] Tomazevic N, Murko E, Aristovnik A. Organizational enablers of artificial intelligence adoption in public institutions: A systematic literature review. Cent Eur Pub Admin Rev. 2024;22:109.

[57] Alserr N, Salepçioğlu MA. Success factors affecting the adoption of artificial intelligence and the impacts of on organizational excellence: A case to be studied in the MENA region, and Turkey in particular. In: International Conference on Business and Technology, 2021.

[58] Lee M, Choi M, Yang T, Kim J, Kim J, Kwon O, et al. A Study on the Advancement of Intelligent Military Drones: Focusing on Reconnaissance Operations. IEEE Access. 2024;12:55964–75. doi: 10.1109/access.2024.3390035

[59] Blei DM, Ng AY, Jordan MI. Latent dirichlet allocation. Journal of Machine Learning Research. 2003;3(Jan):993–1022.

[60] Grootendorst M. BERTopic: Neural topic modeling with a class-based TF-IDF procedure. arXiv preprint. 2022. doi: 10.48550/arXiv.2203.05794

[61] Reimers N. all-MiniLM-L6-v2. https://huggingface.co/sentence-transformers/all-MiniLM-L6-v2. 2021.

[62] Reimers N, Gurevych I. Sentence-bert: Sentence embeddings using siamese bert-networks. arXiv preprint. 2019. doi: 10.48550/arXiv.1908.10084

[63] McInnes L, Healy J, Melville J. Umap: Uniform manifold approximation and projection for dimension reduction. In: 2018. https://arxiv.org/abs/1802.03426

[64] Campello RJGB, Moulavi D, Zimek A, Sander J. Hierarchical Density Estimates for Data Clustering, Visualization, and Outlier Detection. ACM Trans Knowl Discov Data. 2015;10(1):1–51. doi: 10.1145/2733381

[65] Röder M, Both A, Hinneburg A. Exploring the Space of Topic Coherence Measures. In: Proceedings of the Eighth ACM International Conference on Web Search and Data Mining, 2015. 399–408. doi: 10.1145/2684822.2685324

[66] Davis FD, Bagozzi RP, Warshaw PR. User Acceptance of Computer Technology: A Comparison of Two Theoretical Models. Management Science. 1989;35(8):982–1003. doi: 10.1287/mnsc.35.8.982

[67] Engström E, Vartanova I, Viberg Johansson J, Strimling P. What drives acceptance of everyday AI? Explaining the variation in usage across 17 recommender systems. Explaining the Variation in Usage Across. 17.

